# The Michael Reese Hospital, Chicago

**Published:** 1913-07-19

**Authors:** 


### AMERICAN HOSPITALS AND THEIR SYSTEM OF MANAGEMENT.*

### The Michael Reese Hospital, Chicago.

Lest the unwary reader be misled by the title, let us preface this abbreviated notice by saying that the book does not claim to be a compendium of hospital knowledge drawn from world-wide sources and based on the recorded experiences of well-known professional administrators—rather is it the tale of the experience of a Chicago physician and an American architect, who openly lament that there is practically no literature from the pens of "the many splendid hospital administrators" to draw upon for inspiration. We take leave to doubt whether the compilers would not have been better advised to spare themselves this regret by labelling their work with some less pretentious and high-sounding title, and thus at least apprise the reader of the fact that the existence of European hospitals has been almost completely ignored by these worthy collaborators. If a big book (this is a volume of some 600 pages) deserves a big title, the title in this case is justified, otherwise the authors would have been better advised to confine themselves to the equally brief but more veracious appellation "A Modern Hospital." Once the foregoing situation is realised, one is free to state that there is hardly any branch or aspect of an American hospital, or American hospital service, which is not dealt with in the course of the work.

We do not propose on this occasion to treat of the highly technical architectural, surgical, and medical details with which the book abounds, illustrated as they are by numerous designs and reproductions; our criticism will be limited to the general features of the book and its construction.

The introduction deals with three different types of general hospital—viz.: "First, those that are wholly charitable in their good office; second, those that are partly given over to charity and partly to pay-patients; third, those that accept only pay-patients." Treating of the first type of case, our authors are almost brutal in their candour. "So great, indeed, seems to be the change in the public mind within recent years, that it is certain the day is not far distant when the public hospital will become an object of pride to society, where the poor will have the same advantages in the treatment and cure of disease as private patients in more exclusive institutions; indeed, it may frankly be stated that there are even now, thanks to this aroused public conscience, some institutions in several of the States of the American Union to which even well-to-do people seek admission, because of the high order of administration and of their humane and modern methods."

It looks as if public opinion in the States had yet to be awakened to a fuller consciousness of what is due to the less favoured sufferers before the same high level of efficiency and skill that obtains in the United Kingdom could be expected from the general hospitals of America. This at once leads us to what is a most interesting contrast between the evolution of the American and the British hospital systems. There the pay hospital, in active competition with its rivals, furnishes the latest scientific methods in treatment and the highest standard of technical skill; the fact that the institution is run as a "business proposition" merely stimulates the zeal for efficiency and up-to-date methods. Here, as we all know, it is less than fifty years ago that the first hospital for paying patients was incorporated, and to-day all that is newest and best in the medical, surgical, and institutional field comes, not from the pay-hospital, but from the great general hospitals, the bulk of which are charities pure and simple. The crying need of the middle-class patient for pay wards or homes for paying patients, either within or adjacent to and under the control of our voluntary hospitals, is being tardily recognised in this country, whereas the reverse is the case in the United States; there it is the poor man who has the grievance. This fact was handsomely recognised at the recent Oxford Conference, and it is to be hoped that the medical profession and governing bodies generally will not tarry much longer in going to meet the paying patient half-way by arranging to give him a fair share, at a fair price, of the advantages which are at present monopolised apparently by his less fortunate fellow-citizens.

The British hospital administrator will be interested to compare the relative figures given on page 30 of the cost of the various main items which go to make up the cost per day per patient in the American hospital, but the variation in the standard of prices between the two countries must necessarily deprive such a contrast of much of its intrinsic value. In rough figures, the American hospital is stated to expend $2.50 (9s.) per day per patient. Of this expenditure, 30 per cent. is accounted for by provisions, 7 per cent. by surgical and dispensing charges, 10 per cent. by domestic charges, 8 per cent. by fuel, oil, and ice plant, 36 per cent. by salaries and wages, and the balance by establishment, rent, miscellaneous, and management charges.

Over two hundred pages are devoted to architecture and equipment, followed by some generally sound advice on the executive principles upon which a modern hospital should be conducted. The authors pay a tribute to the nursing system adopted in this country in the course of a very interesting attempt to diagnose the reasons underlying the general dissatisfaction with the modern trained nurse in the States. "If the newly graduated medical man must continue to perform practically all the technical service for patients, then an attempt is being made to teach the trained nurse things she will not have use for, and she must delegate herself to a far simpler office, just above that of the menial or maid of all work. If she is to take some of these offices off the hands of the physician, she must be taught to do the work in a workmanlike manner, which she isn't taught to-day. . . . Perhaps at least a partial cure for the unsatisfactory situation in the profession of private graduate nursing is due to the fact that the training schools and the *alumnæ* associations have no control or disciplinary power over their members. England has a lesson for us in this regard."

To American institutional readers and the limited class of philanthropists who are desirous of learning something of what the establishment and maintenance of a modern American hospital imply, the book will be of interest; but to the European public it will possess but little practical value from the general point of view.

A general study of the two books induces us to believe that "The Organisation, Construction, and Management of Hospitals," by Dr. A. Ochsner and Mr. M. J. Sturm (Chicago: Cleveland Press), will be found to contain a more attractive survey of the hospital field and the modern hospital than the work to which the present notice relates.

* *The Modern Hospital.* By J. A. Hornsby, M.D., and R. E. Schmidt, Architect. W. B. Saunders Company, Philadelphia and London.

